# Manifestation of Structure of Electron Bands in Double-Resonant Raman Spectra of Single-Walled Carbon Nanotubes

**DOI:** 10.1186/s11671-015-1213-8

**Published:** 2016-01-05

**Authors:** Yurii Stubrov, Andrii Nikolenko, Viktor Gubanov, Viktor Strelchuk

**Affiliations:** V.E. Lashkaryov Institute of Semiconductor Physics, National Academy of Sciences of Ukraine, 45 Nauky pr., 03028 Kyiv, Ukraine; Department of Physics, Kyiv National Taras Shevchenko University, 64 Volodymyrs’ka str., 01601 Kyiv, Ukraine

**Keywords:** Single-walled carbon nanotube (SWCNT), Graphene, Resonance Raman spectroscopy, Double electron-phonon resonance mechanism (DR), Van Hove singularities, Arc-discharge method, Trigonal warping

## Abstract

Micro-Raman spectra of single-walled carbon nanotubes in the range of two-phonon 2D bands are investigated in detail. The fine structure of two-phonon 2D bands in the low-temperature Raman spectra of the mixture and individual single-walled carbon nanotubes is considered as the reflection of structure of their π-electron zones. The dispersion behavior of 2D band fine structure components in the resonant Raman spectra of single-walled carbon nanotube mixture is studied depending on the energy of excitating photons. The role of incoming and outgoing electron-phonon resonances in the formation of 2D band fine structure in Raman spectra of single-walled carbon nanotubes is analyzed. The similarity of dispersion behavior of 2D phonon bands in single-walled carbon nanotubes, one-layer graphene, and bulk graphite is discussed.

## Background

Single-walled carbon nanotubes (SWCNTs) due to their unique physical and mechanical properties have been extremely investigated during the last two decades. High-strength, electric, and thermal conductivities and biological applicability make them an attractive material for nanotechnology and nanoelectronics, and medicine, as well as biosensors and biocatalyst [1]. All these properties are strongly dependent on nanotube structure, namely how the graphene sheet is twisted up to form the nanotube. Therefore, characterization of SWCNT parameters (*n*, *m*—indices) is always been a priority.

Resonance Raman spectroscopy plays a great role in SWCNT characterization and gives valuable information about their physical and mechanical characteristics. Moreover, it is a powerful tool for investigations of electron and phonon excitations and defects in microstructure of all carbon-based materials [2, 3].

The first-order Raman spectra of such materials always show two main features: the graphite-like G band (~1580 cm^−1^), caused by scattering on Brillouin zone center phonons, and D band (~1350 cm^−1^) whose existence is related with defect-induced resonant scattering [4]. The G bands in the Raman spectra of SWCNTs show a doublet structure (split into G^+^ and G^−^ components). The shape of the G^−^ component is highly sensitive to whether the SWNT is metallic, where a broad structure of the G^−^ band appears, or semiconducting (single-component G^−^ band) [5]. Besides G and D bands, another prominent feature at ~100–400 cm^−1^, called the radial breathing mode (RBM), appears in the first-order Raman spectra of SWCNTs. The frequency position of the RBM modes gives information about distribution of single-walled carbon nanotube diameters according to the relation ω_RBM_ (cm^−1^) = 204/d_t_ (nm) + 27 (cm^−1^) [6]. Then, taking into account the SWCNT diameter distribution and comparing the energy of Raman excitation with that known from Kataura plot optical transition energies E_ii_ (i = 1, 2, 3, …), one can assign the chiral indices (*n*, *m*) of the particular nanotubes excited in the resonant Raman process.

Second-order Raman spectra of carbon-based materials, and particularly of single-layer graphene, are dominated by 2D band at ~2700 cm^−1^, caused by the existence of double electron-phonon resonance (DR) mechanism. The phonons with wave vector *q* = 2*k*, where *k* is an electron wave vector, participate in electron-phonon scattering in DR process, thus making it possible to study the structure of electron bands from the analysis of resonance Raman spectra [7, 8].

Quantum states of 2D graphite (graphene) in a complex way modifies, when transforming into 1D states of the nanotubes leading to appearance of van Hove singularities (vHSs) in a density of electron states (DOS), which determine the electronic properties of SWCNTs. It also results in modification of the selection rules for the DR process [9, 10].

The present paper studies the dispersion of two-phonon 2D bands in the Raman spectra of SWCNTs, measured at liquid nitrogen temperature. The structure of 2D phonon bands is analyzed in relation to the electron band structure of SWCNTs, and possible resonant processes are discussed.

## Methods

### Samples

Single-walled carbon nanotubes used in this study were synthesized by the arc-discharge method in a 600-mbar He atmosphere with nickel and yttrium oxides as catalysts. The diameter distribution of the investigated carbon nanotube mixture was estimated from the experimental frequencies of radial breathing modes (RBM) as d_t_ = 1.51 ± 0.30 nm [11].

### Raman Measurements

Micro-Raman spectra were measured in backscattering geometry at room and liquid nitrogen (77 K) temperatures using triple Raman spectrometer T-64000 Horiba Jobin-Yvon, equipped with cooled CCD detector. Lines of Ar-Kr ion laser with wavelengths of 454.5, 457.9, 476.5, 488.0, 496.5, 514.5, 520.8, 530.9, 568.2, and 647 nm were used for excitation. Excited radiation was focused on the sample surface with 50× optical objective. The laser power on the sample surface was always kept below 1 mW, in order to obtain acceptable signal to noise ratio and to prevent laser heating effect.

## Results and Discussion

**Fig. 1:**
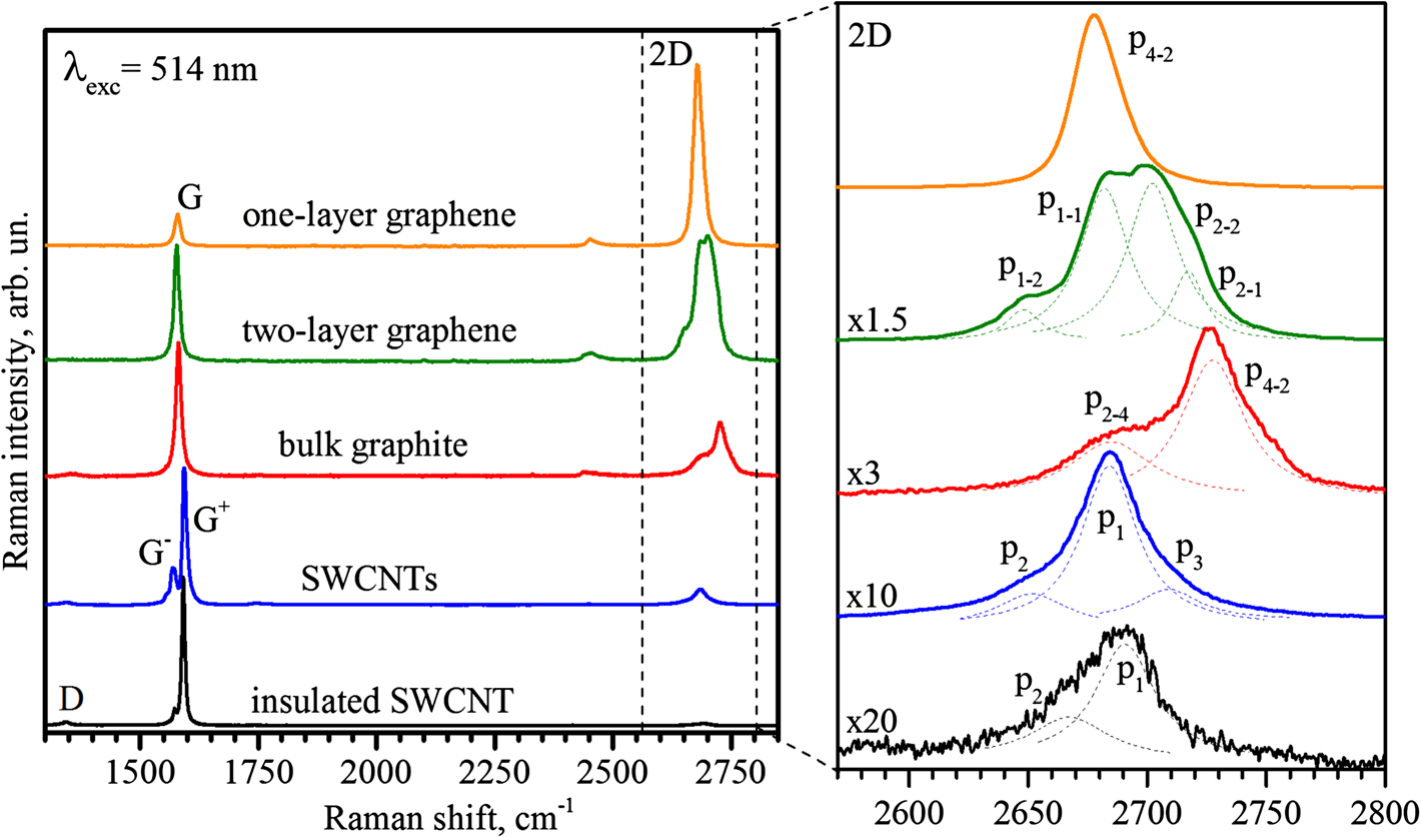
Raman spectra. Raman spectra of single-layer graphene, two-layer graphene, bulk graphite, mixture of SWCNTs, and single SWCNT measured at room temperature; excitation wavelength λ_exc_ = 514.5 nm

Figure 1 shows the Raman spectra of one-layer graphene, two-layer graphene, bulk Bernal graphite with (ab)_n_-stacking, individual SWCNT, and mixture of SWCNTs. In the Raman spectra of one-layer graphene besides allowed in the first-order scattering processes of G band, which is caused by twofold degenerated valence intra-layer vibrations of C-atoms, intensive single two-phonon 2D band is observed [7]. This band is due to second-order processes for phonons near the K-point [12]. The intensity relation of G and 2D bands (I_2D_/I_G_ ≈ 5) and uniform contour of 2D band are the typical features of single-layer graphene [7, 13]. The 2D band in the Raman spectra of two-layer graphene consists of four components, which are well approximated by four Lorentz contours [13]. These components are known to be related with the structure of electron bands, which are revealed due to double electron-phonon resonance [7]. The 2D band in the Raman spectrum of monocrystalline Bernal graphite contains only two components, which also reflects the electron band structure.

As it was mentioned above, 2D band in the Raman spectra of two-layer graphene consists of four components (p_1–2_, p_1–1_, p_2–2_, p_2–1_) (Fig. 1). The interlayer interaction in two-layer graphene leads to an increase of 2D line component number as compared with one-layer graphene, which connects with Davydov splitting (DS) of electron states of one-layer graphene. Thereby, π and π* electron bands of graphene monolayer divide into four, π_1_, π_1_*, π_2_, and π_2_*, bands in the case of graphene bilayer. Dispersion of electronic states near the K-point in such graphene has parabolic behavior instead of linear behavior of one-layer graphene [14].

Taking into account the anisotropy of optical absorption (emission), only electronic transitions ${\displaystyle {\uppi}_1}\rightleftharpoons {\displaystyle {\uppi}_1^{*}}$ and ${\displaystyle {\uppi}_2}\rightleftharpoons {\displaystyle {\uppi}_2^{*}}$ are allowed in bulk graphite crystals.

In bilayer graphene, the anisotropy of optical transitions is less sufficient than for bulk crystalline graphite and transitions ${\displaystyle {\uppi}_2}\rightleftharpoons {\displaystyle {\uppi}_1^{*}}$ and ${\displaystyle {\uppi}_1}\rightleftharpoons {\displaystyle {\uppi}_2^{*}}$ become also allowed [15]. That is why the 2D Raman band consists of two and four distinct components in the case of bulk graphite and two-layer graphene, respectively.

Raman spectra of SWCNT mixture and insulated SWCNT are also shown in Fig. 1. The G band in the Raman spectrum of SWCNTs consists of two components G^−^ (1565.6 cm^−1^) and G^+^ (1590.5 cm^−1^), which are the components of a chiral doublet. The G^+^ band corresponds to valence vibrations of armchair nanotubes perpendicular to nanotube axis and the G^−^ band to valence vibrations of zigzag nanotubes parallel to the nanotube axis [9].

As it can be seen from Fig. 1, the 2D band at 2700 cm^−1^ in the Raman spectrum of isolated SWCNT consists of two components (p_1_, p_2_) and contains three components (p_1_, p_2_, p_3_) in the case of SWCNT mixture. These components are well-resolved and could be approximated by three Lorentz contours. The position of the main p_1_ peak correlates with the position of 2D band of single-layer graphene. The low-energy peak p_2_ is downshifted to ~33 cm^−1^ with respect to the most intensive p_1_ peak, and the position of p_3_ peak is upshifted to ~26 cm^−1^.

**Fig. 2:**
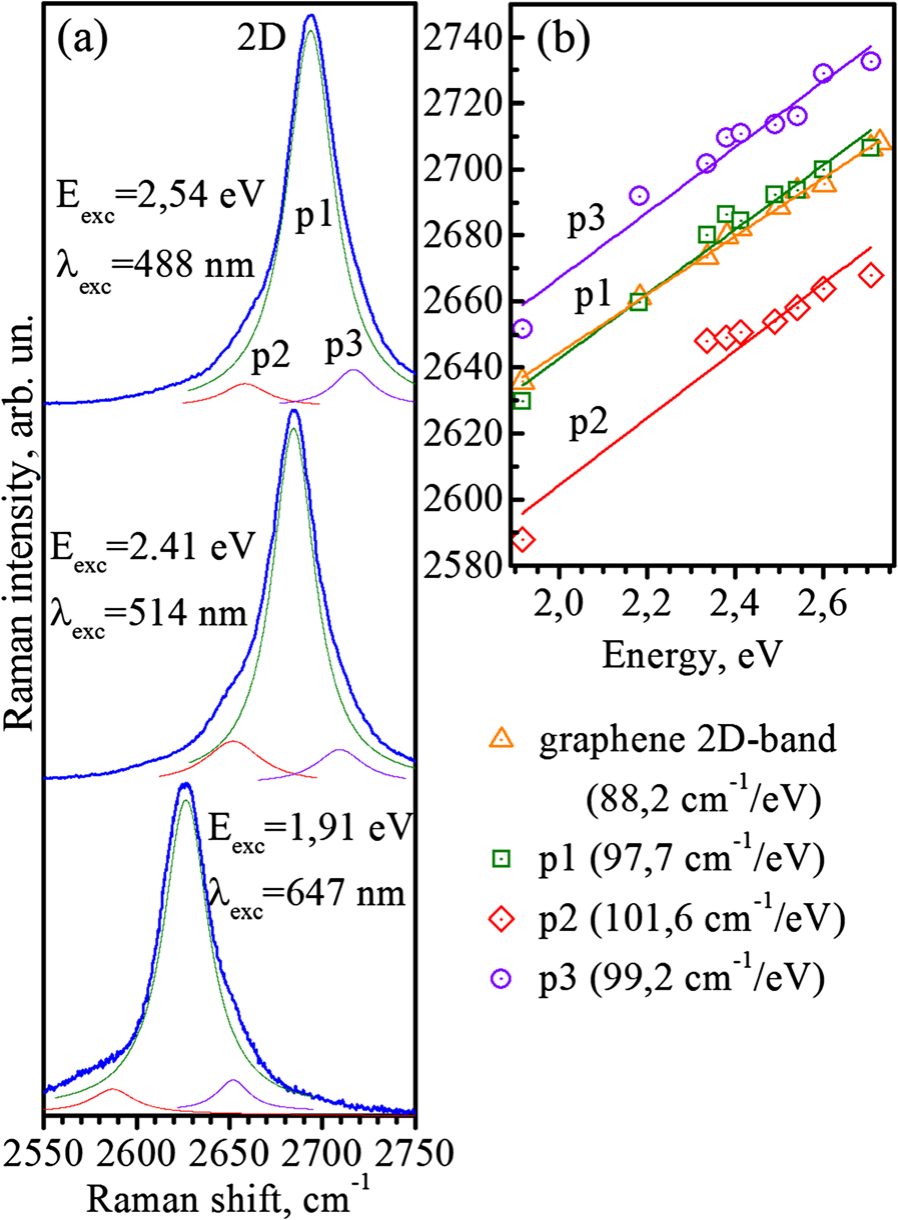
2D Raman band of SWCNT mixture at different excitation energies. Resonant Raman spectra in the range of 2D band for SWCNT mixture (**a**). Dispersion of 2D band components for SWCNTs (**b**). Dispersion of graphene 2D band is also shown for reference. T = 77 K

In order to figure out the nature of the observed structure in 2D Raman band of SWCNTs, we carried out the resonance low-temperature (77 K) Raman measurements. Figure 2 shows the 2D band Raman spectra of SWCNT mixture measured at different excitation energies. As it can be seen, each 2D band in Fig. 2 consists of three components, which can also be approximated by Lorenz contours. Figure 2 also shows the dispersion behavior of the analyzed components as well as the dispersion of 2D band for single-layer graphene [8]. The dispersion of analyzed components appeared to be almost linear with slight variations from the linear behavior. The position and slope of the most intensive p_1_ peak almost coincide with the corresponding values for 2D band of single-layer graphene. The slopes of p_2_ and p_3_ peaks also appeared to be close to the dispersion of single-layer graphene 2D band, with average frequency downshift for p_2_ peak about 36 cm^−1^ and the upshift for p_3_ peak about 26 cm^−1^.

The splitting of 2D band in the Raman spectra of SWCNTs was observed earlier [5, 10, 16] and was explained by the peculiarities of double electron-phonon resonance process providing different resonant conditions for resonances with different van Hove singularities (vHSs) of SWCNTs.

From the analyses of frequency position of observed radial breathing modes (RBM) at used excitation energies, we analyzed the diameter distribution of SWCNTs, which are excited in resonant Raman process [11]. Thus, in the considered resonance process, SWCNTs with diameters in the range of 1.20–1.79 nm are excited. Taking into account the diameter distribution of SWCNTs, the range of used excitations and making the correlation with the Kataura plot in ref. [9], it is possible to conclude that in our case, only the resonant processes with semiconducting E_33_, E_44_, and metallic E_11_ vHSs can be observed. At energies near E_exc_ = 1.91 eV, mostly metallic SWCNTs are involved in resonant process, which is additionally confirmed by the presence of broad G band registered in the Raman spectra (not shown here) [17].

**Fig. 3:**
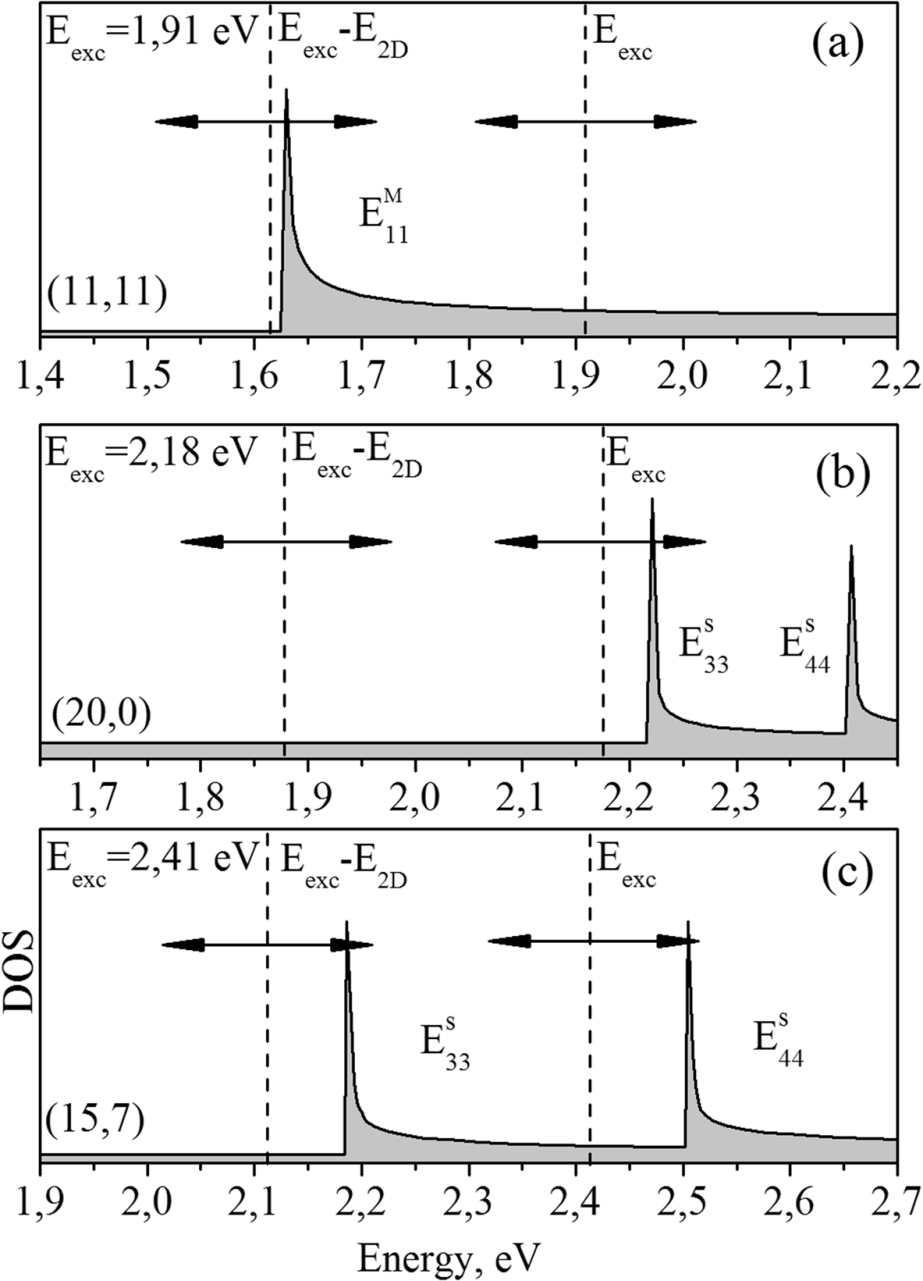
Examples of possible energy schemes illustrating different types of resonances with van Hove singularities (vHSs) giving rise to 2D Raman band of SWCNTs. **a** Incoming and outgoing resonances with ${\mathrm{E}}_{11}^{\mathrm{M}}$ vHSs for armchair (11,11) nanotube at *E*_exc_ = 1.91 eV. **b** Incoming resonance with ${\mathrm{E}}_{33}^{\mathrm{S}}$ vHSs for zigzag (20,0) nanotube at E_exc_ = 2.18 eV. **c** Both incoming and outgoing resonances corresponding with ${\mathrm{E}}_{44}^{\mathrm{S}}$ vHSs and ${\mathrm{E}}_{33}^{\mathrm{S}}$ vHSs for chiral (15,7) nanotube at E_exc_ = 2.41 eV. Horizontal lines with arrows show the width of the resonant window [5, 10]

Figure 3 shows the examples of possible schemes of resonant processes within the double electron-phonon resonance with vHSs of different SWCNTs giving rise to observed features of SWCNTs 2D band [10]. Thus, the most intensive p_1_ peak can be attributed to incoming resonance processes shown in Fig. 3a–c, which in fact is similar to the case of one-layer graphene. The high intensity of p_1_ peak is provided by all types of mentioned resonant processes giving rise to this feature. The nature of less intensive downshifted p_2_ peak can be related to the processes of output resonances with vHSs (Fig. 3a, c). The upshifted p_3_ peak also could be a sequence of outgoing resonant conditions but considering the effect of trigonal warping [5, 16].

From the obtained dispersion behavior of SWCNT 2D band and taking into account the energy of 2D band, which in our case makes ~0.3 eV, the expected downshift of p_2_ peak frequency position with respect to p_1_ peak in the considered double-resonant process can be estimated as ~30 cm^−1^ [10], which is close to experimentally observed downshift for peak p_2_.

The similar dispersion of the most intensive p_1_ peak in the Raman spectra of SWCNTs and single-layer graphene 2D band, as well as almost linear dispersions of additional p_2_ and p_3_ peaks, in our opinion, reflects the general nature of SWCNT electron bands, which originate from single-layer graphene electron bands within the zone-folding procedure, in such a way providing similar conditions for double electron-phonon resonances.

## Conclusions

The fine structure of 2D bands in the Raman spectra of the mixture of SWCNTs and individual SWCNT is explained as caused by the structure of their π-electron zones.

The most intensive p_1_ feature of 2D band in the Raman spectra of SWCNT mixture and individual SWCNT is shown to be related with incoming resonance on SWCNT van Hove singularities, which provides the dispersion behavior with the energy of exciting photons similar to the 2D band of single-layer graphene. The less intensive components of the 2D band (p_2_ and p_3_) in our opinion are caused by additional resonant conditions including the outgoing resonances and trigonal warping effect.

## Abbreviations

DOS: density of electron states
DR: double electron-phonon resonance
DS: Davydov splitting
RBM: radial breathing mode
SWCNTs: single-walled carbon nanotubes
vHSs: van Hove singularities

## References

[1] Burghard M (2005). Electronic and vibrational properties of chemically modified single-wall carbon nanotubes. Surf Sci Rep.

[2] Ferrari AC, Robertson J (2000). Interpretation of Raman spectra of disordered and amorphous carbon. Phys Rev B.

[3] Pimenta MA, Dresselhaus G, Dresselhaus MS, Cançado LG, Jorio A, Saito R (2007). Studying disorder in graphite-based systems by Raman spectroscopy. Phys Chem Chem Phys.

[4] Venezuela P, Lazzeri M, Mauri F (2011). Theory of double-resonant Raman spectra in graphene: intensity and line shape of defect-induced and two-phonon bands. Phys Rev B.

[5] Dresselhaus MS, Dresselhaus G, Hofmann M (2007). The big picture of Raman scattering in carbon nanotubes. Vib Spectrosc.

[6] Michel T, Paillet M, Zahab A, Nakabayashi D, Jourdain V, Parret R, Sauvajol JL (2010). About the indexing of the structure of single-walled carbon nanotubes from resonant Raman scattering. Adv Nat Sci: Nanosci Nanotechnol.

[7] Ferrari AC, Meyer JC, Scardaci V, Casiraghi C, Lazzeri M, Mauri F, Piscanec S, Jiang D, Novoselov KS, Roth S, Geim AK (2006). Raman spectrum of graphene and graphene layers. Phys Rev Lett.

[8] Strelchuk VV, Nikolenko AS, Gubanov VO, Biliy MM, Bulavin LA (2012). Dispersion of electron-phonon resonances in one-layer graphene and its demonstration in micro-Raman scattering. J Nanosci Nanotechnol.

[9] Dresselhaus MS, Dresselhaus G, Saito R, Jorio A (2005). Raman spectroscopy of carbon nanotubes. Phys Rep.

[10] Souza Filho AG, Jorio A, Swan AK, Ünlü MS, Goldberg BB, Saito R, Hafner JH, Lieber CM, Pimenta MA, Dresselhaus G, Dresselhaus MS (2002). Anomalous two-peak *G*′-band Raman effect in one isolated single-wall carbon nanotube. Phys Rev B.

[11] Strelchuk VV, Nikolenko AS, Gubanov VO, Biliy MM, Bulavin LA (2012). Low- and high-frequency intermediate modes with step-like dispersion in resonance Raman scattering of carbon nanotubes. J Nanosci Nanotechnol.

[12] Thomsen C, Reich S (2000). Double resonant Raman scattering in graphite. Phys Rev Lett.

[13] Malard LM, Pimenta MA, Dresselhaus G, Dresselhaus MS (2009). Raman spectroscopy in graphene. Phys Rep.

[14] Partoens B, Peeters FM (2006). From graphene to graphite: electronic structure around the *K* point. Phys Rev B.

[15] Cançado LG, Reina A, Kong J, Dresselhaus MS (2008). Geometrical approach for the study of *G*′ band in the Raman spectrum of monolayer graphene, bilayer graphene, and bulk graphite. Phys Rev B.

[16] Rafailov PM, Maultzsch J, Thomsen C, Dettlaff-Weglikowska U, Roth S (2009). Kohn anomaly and electron-phonon interaction at the K-derived point of the brillouin zone of metallic nanotubes. Nano Lett.

[17] Pimenta MA, Marucci A, Empedocles SA, Bawendi MG, Hanlon EB, Rao AM, Eklund PC, Smalley RE, Dresselhaus G, Dresselhaus MS (1998). Raman modes of metallic carbon nanotubes. Phys Rev B.

